# Postoperative infection, wrong-site surgery, and patient death after elective low-value orthopedic surgery: the epitome of preventable surgical complications

**DOI:** 10.1186/s13037-025-00429-z

**Published:** 2025-03-24

**Authors:** Philip F. Stahel, Navid Ziran

**Affiliations:** 1 Department of Surgery, East Carolina University, Brody School of Medicine, Greenville, NC 27858 USA; 2 Department of Specialty Medicine, Rocky Vista University College of Osteopathic Medicine Parker, CO, USA; 3 Department of Orthopedics, St. Joseph’s Medical Center Phoenix, AZ, USA

**Keywords:** Arthroscopic meniscectomy, Low-value surgery, Postoperative infection, Preventable complications, Patient safety

“Low-value surgery” refers to selected procedures where the expected benefits do not outweigh the risks for harm and complications from the surgical procedure itself, when compared to non-operative treatment options [1, 2]. The risk of unnecessary surgery was recognized more than half a century ago in a 1974 report by Congress outlining that there were annually around 2.4 million unnecessary operations performed on Americans at a cost of US $3.9 billion, and that around 11,900 patients had died in 1974 from unneeded operations [3]. Unlike the analogy of the Food and Drug Administration (FDA) approving pharmaceutical drugs subsequent to a rigorous scientific process, indications for surgical procedures are not subject to any regulatory oversight [4]. A pertinent example of low-value surgery is arthroscopic partial meniscectomy, one of the most commonly performed surgical procedures in the world (also referred to as “meniscus shaving/trimming” or a “knee washout” in lay terms) [5]. This purely elective procedure has come under intense scrutiny in the last decade based on insights from the prospective multicenter, randomized, participant-blinded and outcome assessor-blinded sham/placebo surgery controlled “Finnish Degenerative Meniscal Lesion Study” (FIDELITY) published in 2013 [6]. The FIDELITY trial enrolled 146 middle-age patients of 35 to 65 years of age who were randomized to either arthroscopic partial meniscectomy (*n* = 70) or placebo surgery by knee arthroscopy without meniscectomy (*n* = 76). Patients with absolute indications for surgery due to acute knee trauma or a history of a “locked knee” were excluded from the study [6]. The primary outcome measures were the between-cohort differences in postoperative change from baseline using the “Western Ontario Meniscal Evaluation Tool” (WOMET), Lysholm knee score, and knee pain after exercise at 12 months after surgery [6]. The results of the FIDELITY trial and subsequent follow-up studies demonstrated that the one- to five-year outcomes were no better in patients undergoing a partial meniscectomy versus the sham/placebo surgical procedure alone without meniscal shaving [6–8]. This notion was confirmed by subsequent systematic reviews and meta-analyses of the literature leading to a general consensus that arthroscopic partial meniscectomy in the middle age patient population is reflective of low-value care and rarely indicated or justified compared to conservative measures with symptomatic treatment and physical therapy [9–12].

A recent article published in this journal (www.pssjournal.com) investigated malpractice claims after meniscal surgery from a national Norwegian Patient Registry during a 10-year time-window from 2010 to 2020 [13]. This study sheds further light into the potential sequelae of arthroscopic meniscectomy regarding the impact of postoperative complications from a surgical procedure that may not have been indicated in the first place. The prevalent reason for patients filing a compensation claim was continued knee pain in spite of meniscal surgery [13]. Of larger concern, a total of 98 patients filed a claim for a postoperative infection, which in the realm of arthroscopic meniscectomy implies a high risk for a septic knee joint with potentially devastating short- and long-term consequences [14]. Furthermore, 22 patients claimed a “nerve lesion” which can result in severe functional impairment in case of an intraoperative injury to the deep peroneal nerve [15]. Impressively, 38 patients alleged a “wrong surgical technique” and 16 patients filed a claim for alleged “wrong indication” or “no indication” for surgery (Table 1) [13]. Finally, three patients allegedly sustained a true “never event” related to wrong-site surgery (*n* = 2) and postoperative death (*n* = 1) [13].

These insights confirm the notion that any surgical procedure, whether indicated or not, imposes an imminent underlying risk for severe surgical complications and adverse patient outcomes. Despite the overall low incidence of litigation claims in this study (0.3%), there was an impressive number of 119,528 patients receiving potential low-value surgical care by arthroscopic meniscectomy, of which 372 patients filed a notice of claim [13]. The number of patients who sustained preventable harm from this procedure– in absence of filing a claim– is likely significantly higher. This recent publication in the journal [13] should be a “wake-up call” for surgeons to reconsider surgical indications for elective procedures where the evidence-based literature demonstrates a lack of benefit from surgery compared to non-operative treatment measures.

While arthroscopic meniscectomy in the middle age patient population is easy to track and quantify [5], surgical overtreatment certainly expands to “grey zone” elective indications in other orthopedic domains, including shoulder arthroscopy and spine surgery [1, 2]. Aside from the unjustified risk imposed on patients from a pure patient safety perspective, overtreatment from low-value surgical care also represents a significant driver of preventable health care costs and an incremental burden on the carbon footprint of our planet [16, 17]. Ultimately, as surgeons, we have the ethical and professional duty to remain cognizant of the evolving peer-reviewed literature in our respective area of expertise and to provide valid alternative options to elective surgery within the “shared decision-making” partnership with our patients [18, 19].

**Table 1:** Compensation claim reasons from 372 patients after meniscal surgery from the “Norwegian system of patient injury compensation” database (2010–2020). Adopted with permission under the creative commons attribution 4.0 international license, from: Øhrn FD, Årøen A, Aae TF. Medical negligence compensation claims in knee meniscal surgery in Norway: a cross-sectional study. *Patient Saf. Surg.* 2025, 19:5. (© the authors 2025). Abbreviations: CRPS, chronic regional pain syndrome; DVT, deep vein thrombosis; PE, pulmonary embolism

Compensation claim reasons	Number of patients
Continued knee pain	114
Postoperative infection	98
Wrong surgical technique	38
Impaired knee function or instability	25
Nerve injury	22
Delayed treatment/surgery	21
Wrong surgical indication or no indication	16
Thromboembolic complication (DVT/PE)	13
Inadequate preoperative assessment	10
Iatrogenic injury (not specified)	3
Development of osteoarthritis	2
Knee stiffness	2
Wrong-site surgery (operated on wrong knee)	2
Postoperative compartment syndrome	2
Chronic regional pain syndrome (CRPS)	2
Surgery alleged to initiate rheumatoid arthritis	1
Patient death	1
**Total number of patients**	**372**

## Data Availability

No datasets were generated or analysed during the current study.
